# Supplementary material for: Association between oral microbiome and seven types of cancers in East Asian population: a two-sample Mendelian randomization analysis
Source: Front Mol Biosci. 2023 Nov 21;10:1327893. doi: 10.3389/fmolb.2023.1327893 (PMC10702768; doi:10.3389/fmolb.2023.1327893)
Supplement: Supplementary file 1 [file DataSheet1.ZIP › Supplementary Materials/MR plots of saliva/saliva═╝/Prostate cancer/pheno.2361_to_prostate cancer_forest.pdf]

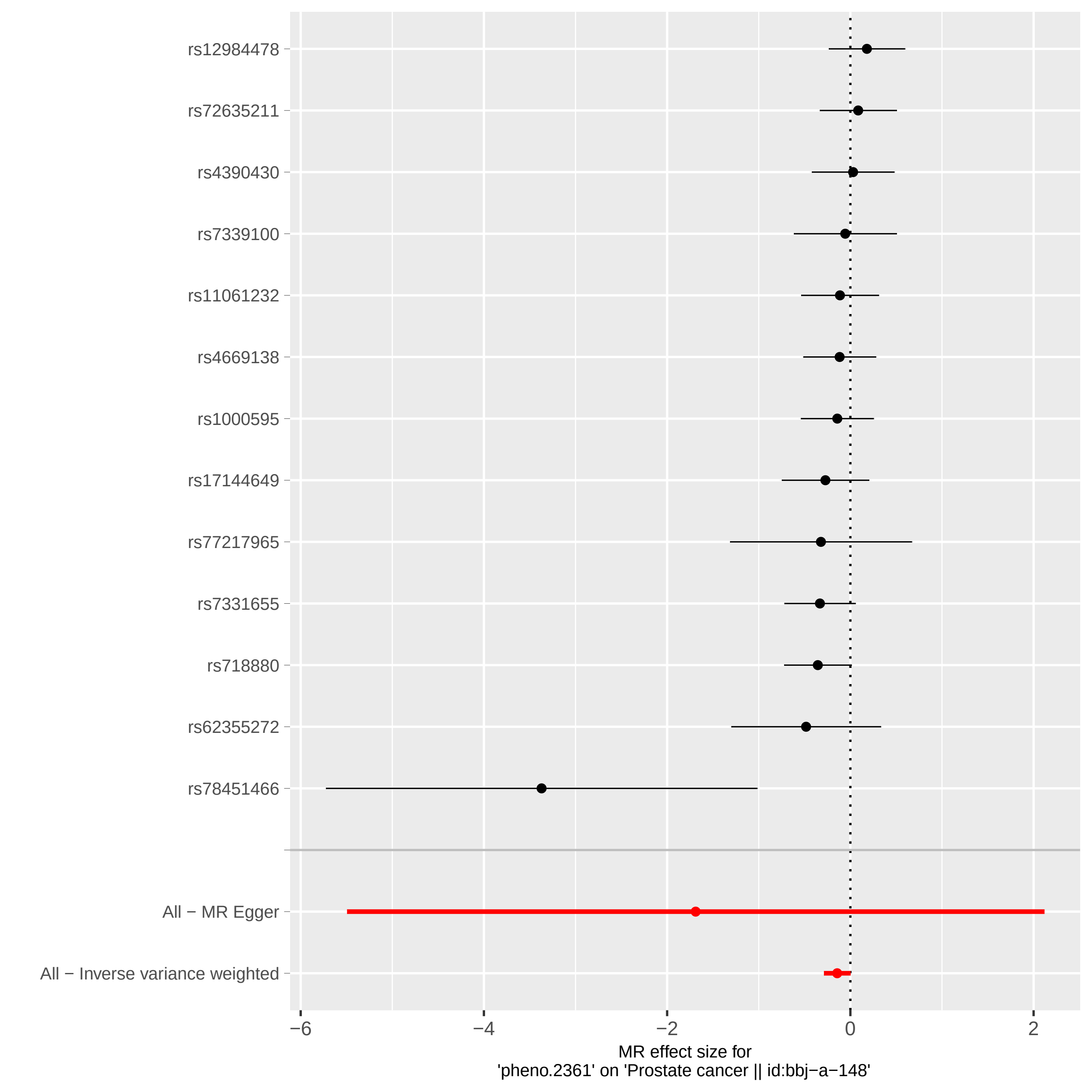
rs12984478
rs72635211
rs4390430
rs7339100
rs11061232
rs4669138
rs1000595
rs17144649
rs77217965
rs7331655
rs718880
rs62355272
rs78451466
All − MR Egger
All − Inverse variance weighted
−6
−4
−2
0
2
MR effect size for
'pheno.2361' on 'Prostate cancer || id:bbj−a−148'
